# Photosynthetic, morphological, and reproductive variations in *Cypripedium tibeticum* in relation to different light regimes in a subalpine forest

**DOI:** 10.1371/journal.pone.0181274

**Published:** 2017-07-12

**Authors:** Bao-Qiang Zheng, Long-Hai Zou, Kui Li, Xiao Wan, Yan Wang

**Affiliations:** State Key Laboratory of Tree Genetics and Breeding; Research Institute of Forestry, Chinese Academy of Forestry, Beijing, China; The National Orchid Conservation Center of China; The Orchid Conservation & Research Center of Shenzhen, CHINA

## Abstract

*Cypripedium tibeticum*, a subalpine orchid species, inhabits various habitats of subalpine forests, mainly including the forest edge (FE), forest gap (FG), and understory (UST), which have significantly different light intensities (FE > FG > UST). However, the ecological and physiological influences caused by different light regimes in this species are still poorly understood. In the present study, photosynthetic, morphological, and reproductive characteristics were comprehensively studied in plants of *C*. *tibeticum* grown in three types of habitats. The photosynthetic capacities, such as the net photosynthetic rate, light-saturated photosynthesis (*P*_max_), and dry mass per unit leaf area (*LMA*), were higher in FE and FG than in UST according to light availability. Compared with FG, the populations in FE and UST suffer from excessively strong and inadequate radiation, respectively, which was further corroborated by the low *Fv*/*Fm* in FE and high apparent quantum yield (*AQY*) in FG. The leaves of the orchids had various proportions of constituents, such as the leaf area, thickness and (or) epidermal hair, to reduce damage from high radiation (including ultraviolet-b radiation) in FE and capture more light in FG and UST. Although the flower rate (*FR*) was positively correlated to both *P*_max_ and the daily mean *PAR*, fruit-set only occurred in the populations in FG. The failures in FE and UST might be ascribed to changes in the floral functional structure and low biomass accumulation, respectively. Moreover, analysis of the demographic statistics showed that FG was an advantageous habitat for the orchid. Thus, *C*. *tibeticum* reacted to photosynthetic and morphological changes to adapt to different subalpine forest habitats, and neither full (under FE) nor low (UST) illumination was favorable for population expansion. These findings could serve as a guide for the protection and reintroduction of *C*. *tibeticum* and emphasize the importance of specific habitats for *Cypripedium* spp.

## Introduction

The genus *Cypripedium* L., which includes 52 species, belongs to the group of slipper orchids (Orchidaceae: Cypripedioideae) [1]. Due to their high ornamental and medical values [2], *Cypripedium* species have been poached ruthlessly in the wild. Moreover, these orchids are experiencing habitat reduction because of biological resource use and agricultural activities, such as deforestation and overgrazing. To date, most *Cypripedium* species have been identified as vulnerable, threatened or endangered in the IUCN Red List of Threatened Species [3]. *C*. *tibeticum* is characterized by a large, broad labellum with a white-margined rim and dark purple flowers; it is distributed in southwest China, Sikkim, Bhutan, and India [1,4]. *C*. *tibeticum* inhabits sparse forests, forest margins, scrubby slopes, and grassy slopes which are found at altitudes of 2,300 to 4,600 m [5]. Although these species are distributed over a wide range of habitats, its populations are suffering a decline throughout the wild [6].

**Fig 1:**
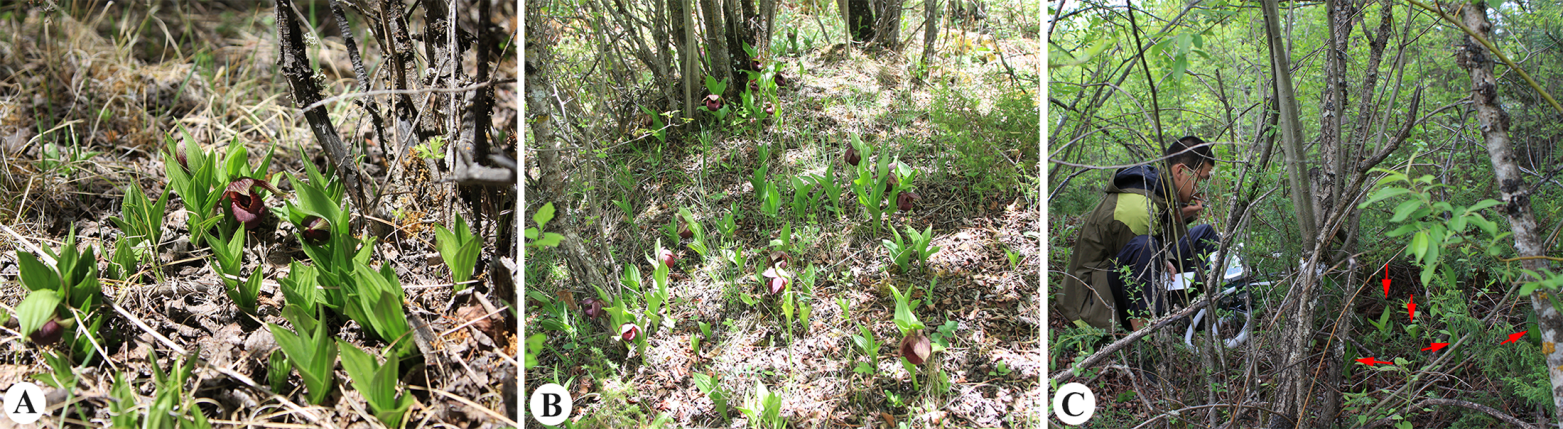
The habitat of *Cypripedium tibeticum* in a subalpine forest. (A) Forest edge. (B) Forest gap. (C) Understory; the red arrows denote individuals of *C*. *tibeticum*.

The landscape of the subalpine forests in southwest China is severely fragmented because of deforestation, degeneration, and climate change [7]. Therefore, the habitats of *Cypripedium* in the fragmented forests have also suffered severe destruction [8] and experienced changing ecological conditions, such as solar radiation, temperature, and humidity changes [9]. Neo-environments can induce morphological and physiological changes in plants [10], which impact their reproductive capacity [11] and survival [12]. Previous studies have suggested that the growth and reproduction of *Cypripedium* are mainly affected by habitat [13–15]. In subalpine mixed deciduous forests, *C*. *tibeticum* is perched in three types of microhabitats, including the forest edge (FE; Fig 1A), forest gap (FG; Fig 1B), and understory (UST; Fig 1C). However, the adaptive strategy of *C*. *tibeticum* to these habitats remains to be evaluated.

Light is a significant factor that drives growth and fitness of plants via photosynthesis. Photosynthetic indexes can help identify suitable growth conditions and plant adaptation strategies to different environments [16,17]. Several studies have been performed on *Cypripedium* species to investigate the effects of leaf anatomical structures [18,19], foliar age [20], light gradients [8,21], and transplant conditions [22] on photosynthesis rates. These studies indicated that some *Cypripedium* species have photosynthetic and morphological plasticity in response to various light regimes or habitats. Other studies assessed the relationships between photosynthesis and reproductive traits [15]. Interestingly, Zhang et al. [15] found that natural populations of *C*. *flavum* have a positive correlation between the percentage of fruiting and quotient of the daily mean photosynthetic rate to light saturated photosynthesis.

In addition to the vegetative morphology, the characteristics of reproductive organs should also be evaluated. Previous studies have shown that *Cypripedium* flowers in greenhouses are always smaller than those in the wild [23], suggesting that the flower structure can be modified by different environments. *C*. *tibeticum* have one-way trap flowers with an easy entrance into the labellum from the front, but an easier exit to the rear [2,5]. In the “escape route”, the distances between the stigma and bottom of the labellum (*SL*) and that between the anther and bottom of the labellum (*AL*) are two key characteristic parameters for the successful pollination of these species. Bumblebee queens (*Bombus lepidus*) are the major pollinators of orchids because they have a body size that is slightly larger than both *SL* and *AL* and can carry off pollen and touch the stigma [5,24]. However, it is still not clear whether the flower structure of *C*. *tibeticum* is modified by their habitat (FE, FG, and UST) and whether the modified flower structures, if any, are related to reproductive success.

In the present work, we investigated the photosynthetic, morphological, and reproductive traits of *C*. *tibeticum* inhabiting three types (FE, FG, and UST) of subalpine forest habitats that have different light availabilities. Our aims were to identify 1) how species adapt to subalpine forest environments by assessing photosynthesis and leaf morphology changes; 2) divergent reproduction in relation to these natural habitats. The results of this study will assist in developing conservation strategies for *C*. *tibeticum* as well as other species of *Cypripedium*.

## Materials and methods

### Ethics statement

The present study was performed in the natural reserve of *Cypripedium* spp. of Shangri-la Alpine Botanical Garden, Yunnan, China. Permissions to enter the reserve and to collect the samples were issued by respective authorities and Prof. Zhendong Fang, the director of Shangri-la Alpine Botanical Garden. We guaranteed no vegetation deterioration in experimental regions during the study.

### Experimental sties and plant materials

The study sites are located in the natural reserve of *Cypripedium* spp. of Shangri-la Alpine Botanical Garden in Northwest Yunnan, China (28°16.732N, 99°10.566E, alt. 3027 m). The mean annual temperature in this region is 5.9°C, with a monthly mean temperature that ranges from -0.4°C to 13.3°C, and this region has 123.8 frost-free days annually. The annual precipitation and evaporation in this region are 648.6 mm and 616.8 mm, respectively. The annual insolation duration in this region is 2155.9 hours, and the annual average relative humidity is 69% (data are from the Meteorological Station of Diqing Prefecture, which is located approximately three kilometers from the experimental sites).

At the study sites, *C*. *tibeticum* has an approximately six-month growth period from late April to September and grows in mountain brown soil, and these sites have a similar water availability to forest, where *Populus yunnanensis* is the dominant species. *C*. *tibeticum* is mainly distributed in three habitats, including FE, FG, and UST. The cohabiting species of *C*. *tibeticum* are *Crataegus chungtienensis*, *Salix rehderiana*, *Sabina squamata*, and *Berberis jamesiana* in UST; *Quercus senescens*, *Euphorbia nematocypha*, *Erigeron breviscapus*, *Epipactis mairei*, and *Anemone rivularis* in FG; and *Salix rehderiana* and *Sabina squamata* in FE. In each habitat, three 4×4 m experimental quadrats were tagged so that successive observations could be made, and the altitude intercept between the lowest- and the highest- quadrat was less than 15 meters.

### Leaf photosynthesis measurements

All diurnal gas exchange rate measurements were made on the second fully expanded leaf counted basipetally from 0700 to 1900 HR (time, 07:00 to 19:00) on clear days during the flowering phase (early June 2011). After equilibration with the local ambient conditions of each habitat, the net photosynthetic rate (*P*_n_), transpiration rate (*E*), stomatal conductance (*g*_s_), intercellular CO_2_ concentration (*C*_i_), atmospheric CO_2_ concentration (*C*_a_), leaf temperature (*T*_l_), and photosynthetic active radiation (*PAR*) were measured with a Li 6400 portable photosynthesis analysis system (Li-COR, Lincoln, NE). According to diurnal measurements, stomata limitation (*L*_s_) was defined as *L*_s_ = 1—*C*_i_/*C*_a_ [25]. Moreover, we obtained additional diurnal *PAR* data from other clean days to describe the light regimes in different habitats. These days included May 18^th^ and September 9^th^ in 2012 and May 20^th^ and September 13^th^ in 2013. At the highest solar altitude, the ultraviolet-B (UV-B) intensity in these quadrats was determined with a UV340B ultraviolet detector (Sanpometer Co. Ltd., Shenzhen, Guangdong, China).

Photosynthetic response curves to light and CO_2_ were also measured. The light response curves were measured from 0700 to 1100 HR. The parameters in the leaf chamber were a CO_2_ concentration of 350 μmol mol^-1^, temperature of 25°C, and relative humidity of atmosphere. The values of *P*_n_ were recorded at the following photosynthetic photon flux densities: 1200, 1000, 800, 600, 400, 200, 150, 100, 80, 60, 40, 20, and 0 μmol m^-2^ s^-1^. The light compensation point (*LCP*), light saturation point (*LSP*), light-saturated photosynthesis (*P*_max_), and apparent quantum yield (*AQY*) were calculated by fitting a modified rectangular hyperbolic model to the observed light response curves as described previously [26].

The CO_2_ photosynthetic response curves were determined from 0700 to 1100 HR in the leaf chamber at a *PAR* of 600 μmol m^-2^ s^-1^, 20°C, and relative humidity of atmospheric. The *P*_n_ values were recorded at the following concentration gradients of CO_2_: 50, 80, 100, 120, 150, 200, 400, 600, 800, 1000, 1200, and 1500 μmol mol^-1^. The CO_2_ compensation point (*CCP*), carboxylation efficiency (*CE*), and maximum RuBP saturated rate of carboxylation (*V*_cmax_) were calculated from the observed response curves using a modified rectangular hyperbolic model [26].

Chlorophyll fluorescence of *C*. *tibeticum* was measured with a Handy PEA chlorophyll fluorimeter (Hansatech Instruments Ltd., King's Lynn, Norfolk, UK). *Fm/Fv* was used to estimate the photoinhibition of PII. The measurements were conducted in the early morning (approximately 0800–1000 HR), noon (approximately 1200–1400 HR), and late afternoon (approximately 1600–1900 HR). To avoid any deviations caused by delays among measurements, repetitive tests were performed on randomly selected plants in the three types of habitats.

### Morphological traits

Pieces from the middle region of the leaf blade were fixed in FAA (formalin, glacial acetic acid, 75% ethanol). After dehydration in gradient ethanol, the samples were cleared in xylene and embedded in paraffin; the paraffin sections were cut using a Microm HM 315 rotary microtome (MICROM International GmbH, Rhein-Neckar-Kreis, Baden-Württemberg, Germany) and mounted on microslides. Leaf (*TtL*) and mesophyll (*TM*) thicknesses were examined and photographed using a Nikon Eclipse E800 light microscope (Nikon Corp., Konan, Minato-ku, Japan) with Image Pro-Plus 6.0 (Media Cybernetics, Inc., Rockville, Maryland, USA) on a workstation running on Windows. The percentage of mesophyll in the leaf thickness (*PML*) was calculated by *TM*/*TtL*. Moreover, small sections were also cut from the middle region of the blade and examined under a SZM-0745T2 stereoscopic microscope (Seepex Co. Ltd., Shenzhen, Guangdong, China). The numbers of adaxial glandular hairs (*NTF*) and abaxial glandular hairs (*NTB*) were counted in ten horizons from the first- and second-last leaves in the three habitats.

Fifteen flowering plants were randomly sampled from UST, FG, and FE. The essential vegetative traits were measured, including the height of the plant (*PH*) and leaf area (*LA*) of the second leaf from the top. These lengths were measured with digital calipers to the closest 0.1 mm and *LA* by using a LA-S plant leaf area analysis system (Wseen Detection Technology Co. Ltd., Hangzhou, Zhejiang, China). The dry mass per unit leaf area (*LMA*) was calculated from leaves collected in June and September after drying at 60°C for 48 hours.

### Demography and reproductive characteristics

The population statistics in each quadrat were investigated from 2011 to 2013. We also adopted shoot-counting to describe the population sizes. The flowering- (*FR*) and fruiting- rates (*FrR*) were estimated as the ratios of the number of flowering shoots to total shoots and fruit to total flowers in each quadrat, respectively. It is important to note that all of the populations in the present study were fertile, which was verified by hand-pollination. The essential floral traits were also measured, including the distance between the anther and bottom of the labellum (*AL*), height between the stigma and bottom of the labellum (*SL*), and width (*LW*) and length (*LL*) of the labellum.

### Statistical analysis

Statistical analyses were performed with SPSS 19.0 (IBM Corp., Armonk, New York, USA) for Windows. Differences among morphological variables were determined using ANOVA and the LSD test for multiple comparisons. Differences were considered significant at *P* < 0.05.

## Results

### Light regimes and diurnal changes of the gas exchange traits

**Fig 2:**
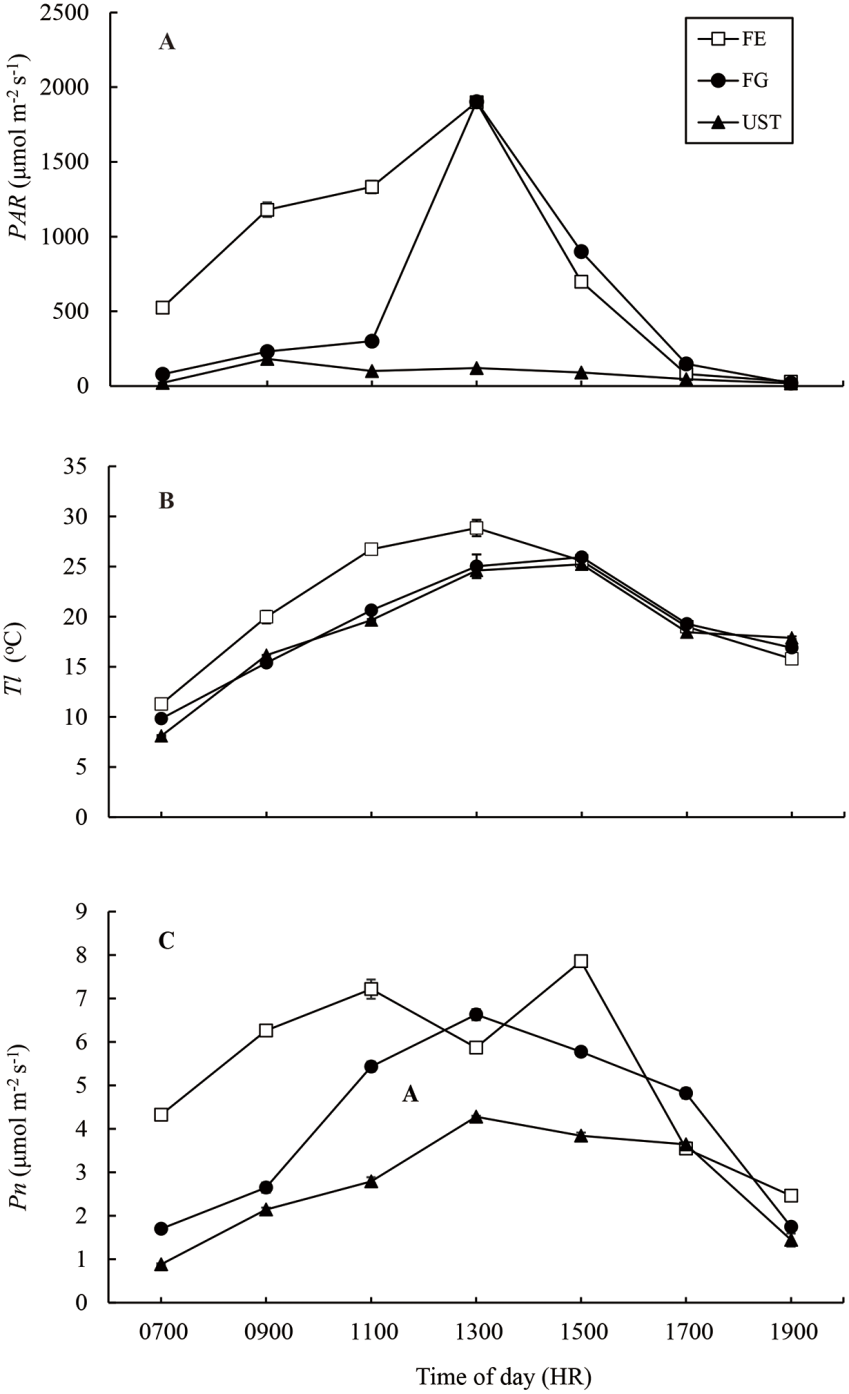
Diurnal changes in photosynthetic active radiation (*PAR*, A), leaf temperature (*T*_l_, B), and net photosynthetic rates (*P*_n_, C) of *C*. *tibeticum* in different habitats. Each point represents the mean ± SE (n ≥ 10).

As expected, the diurnal variations of light showed significant differences among the three habitats. FE had the highest light levels during the day in comparison with FG and UST (Fig 2A). The *PAR* of FG started as high as 500 μmol m^-2^ s^-1^ at 0700 HR and increased rapidly, with the maximum value at 1300 HR, after which it decreased gradually. By contrast, the *PAR*s of both FG and UST remained at a much lower level than FE before 1100 HR, and the *PAR*s differed between FG and UST. The *PAR* in FG showed a peaked value at 1900μmol m^-2^ s^-1^ at approximately 1300 HR. However, *PAR* in UST showed weak fluctuations, varying between 22 and 180 μmol m^-2^ s^-1^ throughout the day. Identical light regimes were also found in May and September (S1 Fig and S1 Table) of 2012 and 2013. The UV-B records at noon are listed in Table 1 and S2 Table. FE had the strongest UV-B radiation and UST had the lowest.

**Table 1:** UV-B radiation (W m^-2^) of the three habitats at noon.

Month	FE	FG	UST
May	49.141±3.566a	12.733±1.701b	0.231±0.299c
September	45.860±2.785a	10.015±1.040b	0.024±0.007c

Each point represents the mean ± SE (n = 6).

In line with the light intensities, *T*_l_ at FE was significantly higher than those of FG and UST before 1300 HR (Fig 2B). However, no differences in leaf temperature were observed between FG and UST, although their light levels were quite different (Fig 2A).

The diurnal variation of *P*_n_ of *C*. *tibeticum* in different habitats is shown in Fig 2C. Overall, plants at FE had the highest *P*_n_, while those at UST had the lowest. The *P*_n_ diurnal curve of plants at FE was bimodal at 1100 and 1500 HR, with a clear midday depression of photosynthesis at 1300 HR (Fig 2C). By contrast, the *P*_n_ diurnal curves of FG and UST were unimodal, with *P*_max_ at 1300 HR. *P*_max_ of plants in FE, FG, and UST were 7.86, 6.63, and 4.28μmol m^-2^ s^-1^, respectively.

**Fig 3:**
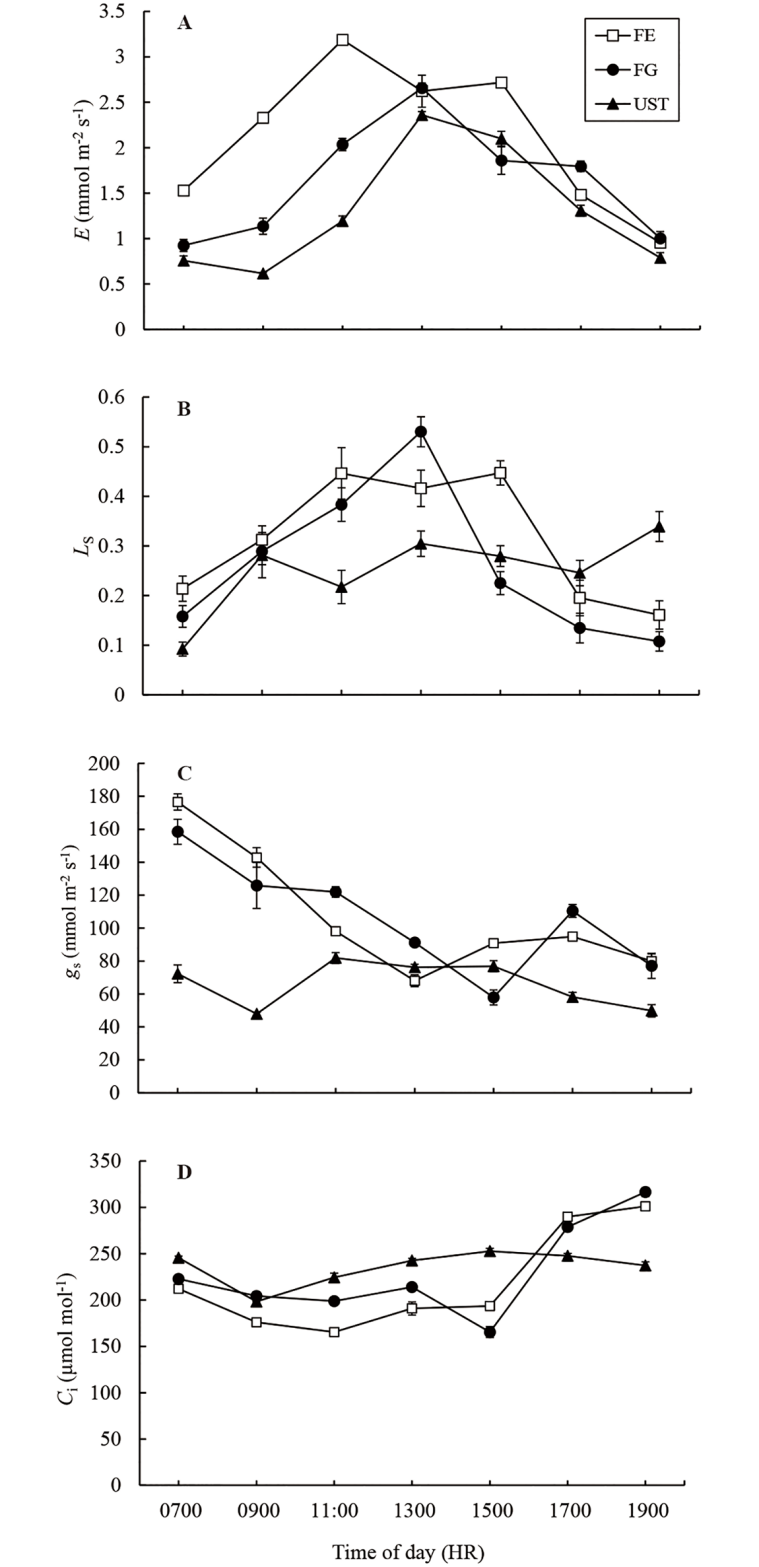
Diurnal changes in the transpiration rate (*E*, A), stomatal limitation value (*L*_s_, B), stomatal conductance (*g*_*s*_, C) and intercellular CO_2_ concentration (*C*_i_, D) in *C*. *tibeticum* in different habitats. Each point represents the mean ± SE (n ≥ 10).

The diurnal changes of *E* at the three sites synchronized with the diurnal dynamics of the leaf photosynthetic rates (Fig 3A v.s. Fig 2C). Similarly, plants in FE had the highest *E*, while those in UST had the lowest. The *g*_s_ of FE and FG showed similar diurnal variations without significant differences, and both decreased gradually in FE and FG from 0700 to 1300 HR and thereafter remained relatively constant (Fig 3C). The *g*_s_ of UST was significantly smaller than those of FE and FG before 1100 HR, after which the *g*_s_ of UST increased slightly to a similar level as those of FE and FG. As a result of the interaction between *P*_n_ and *g*_s_, the *C*_i_ of UST plants was highest before 1500 HR, while the *C*_i_ of FE plants was lowest (Fig 3D). After 1500 HR, the *C*_i_ of FE- and FG- plants increased and were higher than that of UST plants. The variation in *C*_i_ compared with *C*_a_ was used to quantify the stomatal limitation (Fig 3B). The stomatal limitation peaked at approximately 1300 HR for plants of all three habitats. Although differences were found between the three habitats, no significant differences were detected.

**Fig 4:**
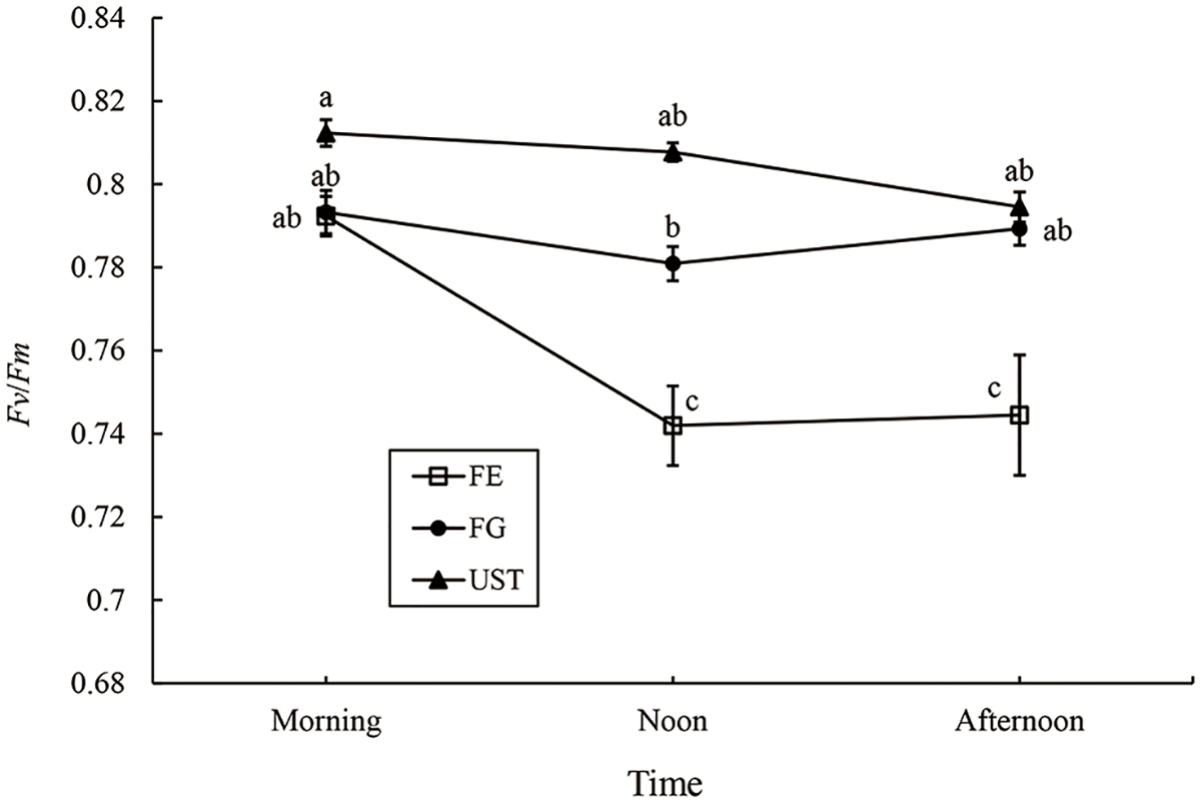
*Fv*/*Fm* of *C*. *tibeticum* in different habitats. Each point represents the mean ± SE (n ≥ 15).

There were no significant *Fv*/*Fm* differences during the day in both FG and UST (Fig 4). Moreover, the parameter in FE had a high level in the morning as in the other two habitats, but declined sharply to a low value at noon and remained at a low level throughout the afternoon.

### Photosynthetic responses to *PAR* and CO_2_

**Fig 5:**
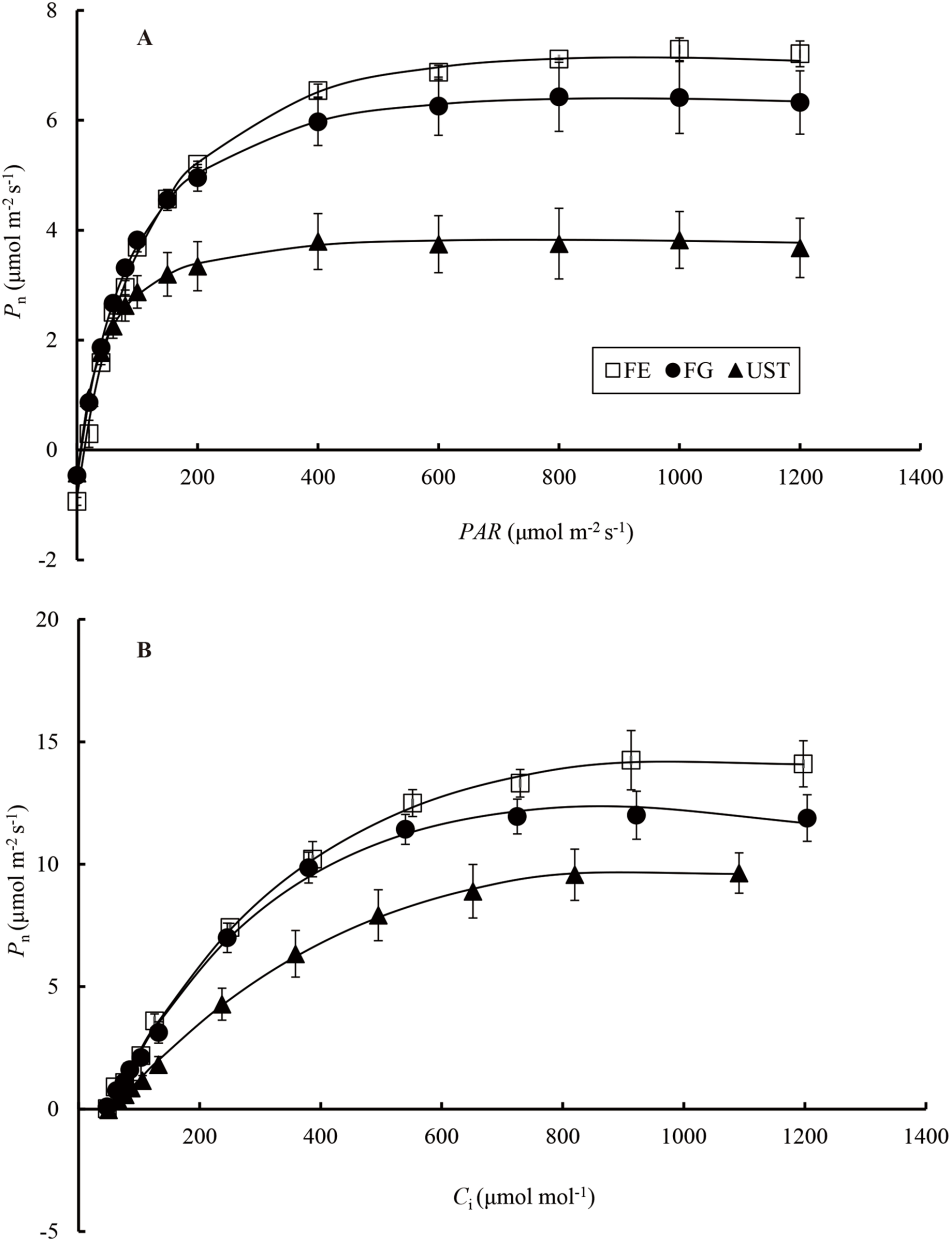
Photosynthetic responses of *C*. *tibeticum* in different habitats to *PAR* (A) and CO_2_ (B). Each point represents the mean ± SE (n = 3).

The photosynthetic responses of *C*. *tibeticum* to *PAR* differed significantly among plants from the three habitats (Fig 5A). Based on these response curves, *LCP*, *LSP*, *P*_max_, and *AQY* were estimated (Table 2). Plants at FE had an *LCP* value (Table 2) that was at least two-fold higher than those at FG and UST. Likewise, significant differences were observed for *LSP* and *P*_max_ and followed the pattern: FE>FG>UST. Moreover, there were significant differences in *AQY* between UST and the other two habitats (Table 2).

**Table 2:** Leaf gas exchange parameters of *C*. *tibeticum* in different habitats.

Parameters	Forest edge	Forest gap	Understory
*AQY* (μmol m^-2^ s^-1^)	0.086±0.002b	0.0874±0.004b	0.1035±0.004a
*CCP* (μmol/mol)	49.16±1.45a	51.18±3.17a	55.70±1.07a
*CE* (μmol m^-2^ s^-1^)	0.061±0.007a	0.063±0.007a	0.031±0.004b
*LCP* (μmol m^-2^ s^-1^)	12.98±1.12a	6.20±0.66b	4.61±0.58b
*LSP* (μmol m^-2^ s^-1^)	987.6±97.6a	889.7±96.8ab	655.1±42.6b
*P*_max_ (μmol m^-2^ s^-1^)	7.15±0.07a	6.42±0.60a	3.81±0.95b
*R*_d_ (μmol m^-2^ s^-1^)	1.002±0.102a	0.508±0.052b	0.437±0.070b
*R*_l_ (μmol m^-2^ s^-1^)	2.723±0.316a	2.883±0.101a	1.657±0.170b
*V*_cmax_ (μmol m^-2^ s^-1^)	14.27±0.98a	12.39±0.76ab	9.80±0.96b

Mean ± SE (n ≥ 15); *AQY*, apparent quantum yield; *CCP*, CO_2_ compensation points; *CE*, carboxylation efficiency; *LCP*, light compensation point; *LSP*, light saturation point; *P*_max_, light- saturated photosynthetic rate; *Rd*, dark respiration rate; *R*_l_, photorespiration rate; *V*_cmax_, maximum RuBP saturated rate of carboxylation. Different letters in the same row indicate significant differences (*P* < 0.05).

Similar to the light response curve, the photosynthetic responses of *C*. *tibeticum* to CO_2_ also differed significantly among plants from the three habitats (Fig 5B and Table 2). For a given *C*_i_ higher than 200 μmol mol^-1^, *P*_n_ of FE plants was highest, followed by *P*_n_ of FG and UST plants. *CCP* of plants in the three groups varied from 41.18 to 57.29 μmol mol^-1^ without significant differences (*P* > 0.05, Table 2). The *CE* were not significantly different between FE and FG, but these were higher than that of UST. The *R*_d_ and *V*_cmax_ showed higher values in sites with more illumination. Although the photorespiration rate (*R*_l_) had no significant differences between FE and FG, it was the lowest in UST.

### The morphological and reproductive traits

#### Morphological traits

**Fig 6:**
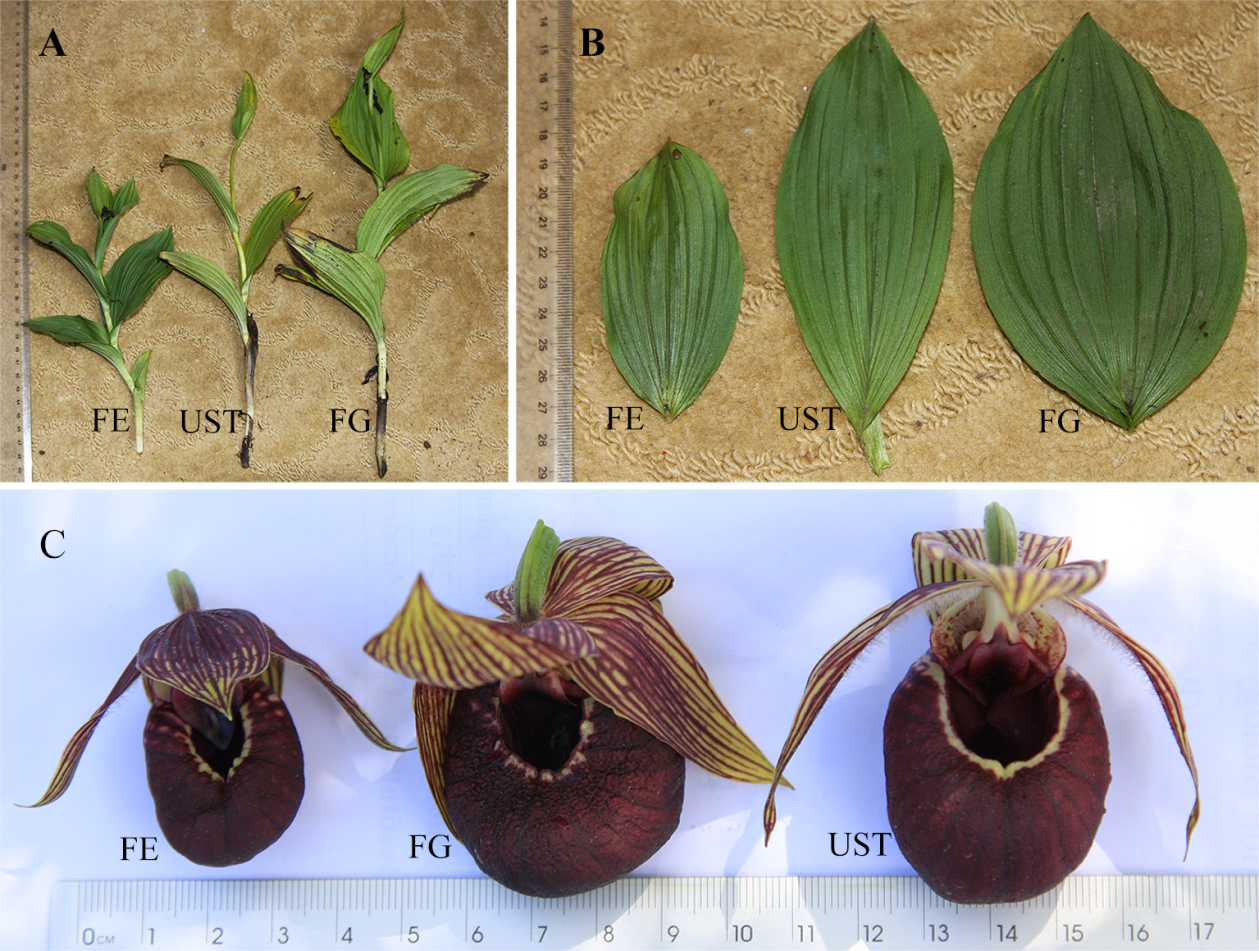
Plants (A), leaves (B), and flowers (C) of *C*. *tibeticum* in the forest edge (FE), forest gap (FG), and understory (UST) in the subalpine forest.

**Fig 7:**
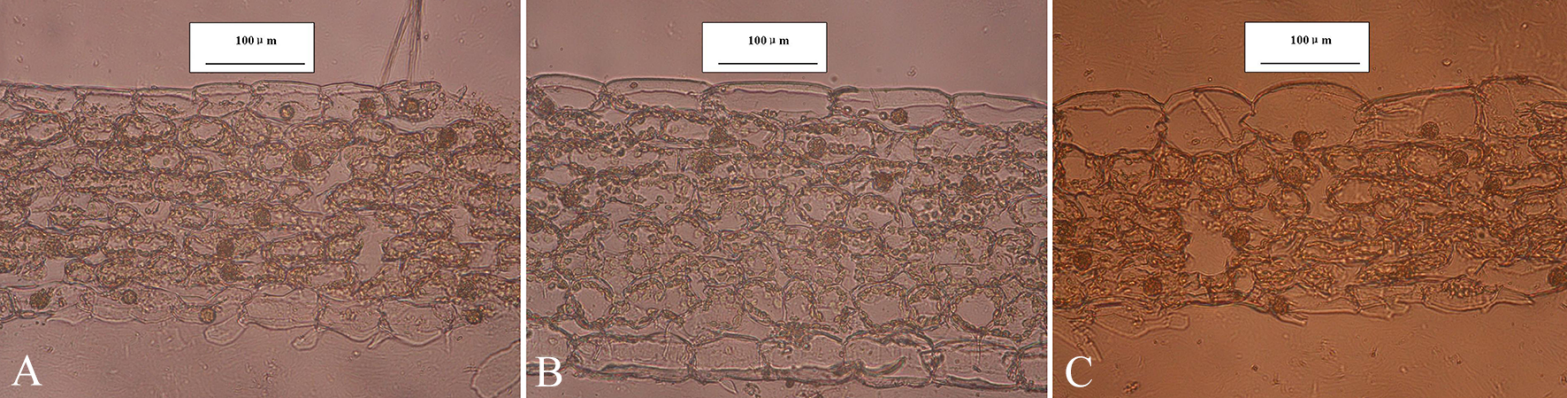
Leaf cross section of *C*. *tibeticum* in FE (A), FG (B), and UST (C). The bar is 100 μm.

Plants were vigorous and tall in FG, slim and tall in UST, and short in FE (Table 3; Fig 6A). The leaf characteristics of *C*. *tibeticum* varied significantly with their habitat types. *LA* in FG was highest, intermediate in UST, and lowest in FE (Fig 6B, Table 3). Leaf anatomical analysis revealed that the leaf of *C*. *tibeticum* was the isolateral type without palisade cells and a spongy parenchyma (Fig 7). The mesophyll cells of leaves in FE and FG were closely spaced and had few chloroplasts, but those in UST were arranged loosely and had fewer chloroplasts. Moreover, differences were found for most of the other anatomical parameters (Table 3), such as the thickness of leaf tissues and densities of glandular hairs on the epidermis. The thicknesses of the blade, mesophyll, and hypodermis were significantly different, with the thickest found in FE and the thinnest in UST. However, plants in UST had the thickest adaxial epidermis among the three habitats. The adaxial leaf epidermis of the population in FE was the thinnest and were as thin as their abaxial epidermis. The percent of mesophyll in leaf thickness ranged from 57.24% to 76.71%, and these values were maximum in plants in FE and minimum in plants in UST. Glandular hairs on the leaf were mainly distributed on the abaxial epidermis. Plants in FE had more glandular hairs than those in FG and UST (*P* < 0.05), although the differences were not significant between FG and UST. Moreover, *LMA* of *C*. *tibeticum* was highest in FG, lowest in UST, and showed an intermediate value at FG.

**Table 3:** Plant heights and leaf traits of *C*. *tibeticum* in different habitats.

Parameters	FE	FG	UST
*PH* (cm)	16.24±0.54b	26.39±0.86a	25.16±0.52a
*TtL* (μm)	155.16±1.47b	194.52±0.96a	143.58±1.14c
*TM* (μm)	114.50±1.32b	141.71±2.01a	85.82±1.32c
*PML* (%)	74.41±0.52a	73.23±0.69a	59.86±0.58b
*LMA* (June, g m^-2^)	36.67±0.51a	32.93±0.24b	28.43±0.45c
*LMA* (September, g m^-2^)	42.69±0.80a	41.88±1.06a	34.44±0.92b
*LA* (cm^2^)	34.50±1.02c	58.34±1.80a	44.43±0.82b
*NTB* (cm^-2^)	25.09±0.98a	15.11±0.89b	15.21±1.43b
*NTF* (cm^-2^)	57.20±1.27a	38.13±0.78b	37.21±2.16b

Values are the mean ± SE (n ≥ 15). *PH*, plant height; *TtL*, leaf thickness; *TM*, thickness of the mesophyll; *PML*, percentage of mesophyll in leaf thickness; *LMA*, dry mass per unit leaf area; *LA*, area of the second leaf; *NTB*, abaxial glandular hairs; *NTF*, number of adaxial glandular hairs. Different letters in the same row indicate significant differences (*P* < 0.05).

#### Reproductive traits

**Fig 8:**
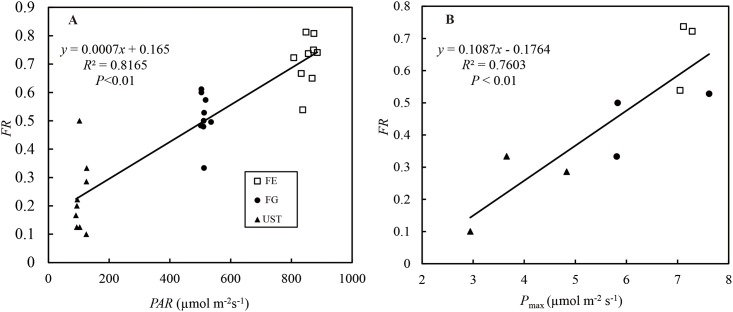
Effects of the (A) daily mean *PAR* and (B) *P*_max_ on the flowering rate (*FR*) of *C*. *tibeticum* in subalpine forest habitats.

The floral functional traits, *LL*, *LW*, *SL*, and *AL*, of the plants in FE were apparently smaller than those in the other two habitats, but no significant differences were observed between plants in FG and UST (Table 4, Fig 6C). For three consecutive years, we recorded the flower rates (Table 5) of considerable species in the three habitats. There were significant differences in the flower rates at different sites. The populations in FE had the highest flowering rate, those in FG had an intermediate flowering rate, and those in UST had the lowest flowering rate. Regression analysis revealed that there was a positive linear correlation between *P*_max_ and *FR* (Fig 8A). A positive correlation between the daily mean *PAR* and *FR* was also observed (Fig 8B). As for the fruiting rates, only *C*. *tibeticum* in FG set fruits with low values of 12.2 ± 2.0%, and no fruit setting occurred in the other two habitats (Table 4).

**Table 4:** Floral functional morphology of *C*. *tibeticum* in different habitats.

Habitats	FE	FG	UST
*LL*(cm)	4.27±0.23b	4.79±0.39a	4.90±0.08a
*LW* (cm)	2.67±0.21b	4.00±0.71a	3.80±0.23a
*SL* (cm)	0.54±0.067b	0.70±0.057a	0.69±0.020a
*AL* (cm)	0.43±0.054b	0.56±0.045a	0.55±0.016a

Values are the mean ± SE (n ≥ 15). *LL*, length of the labellum; *LW*, width of the labellum; *AL*, distance between the anther and bottom of the labellum, *SL*, height between the stigma and bottom of the labellum. Different letters in the same row indicate significant difference (*P* < 0.05).

**Table 5:** Reproduction traits of *C*. *tibeticum* from 2011 to 2013 in different habitats.

Habitats	FE	FG	UST
*FR* (%)	71.4±2.8a	51.2±2.8b	22.9±4.3c
*FrR* (%)	0b	12.1±2.0a	0b

Values are the mean ± SE (n = 9). *FR*, flowering rate; *FrR*, fruiting rates; different letters in the same row indicate significant difference (*P* < 0.05).

**Fig 9:**
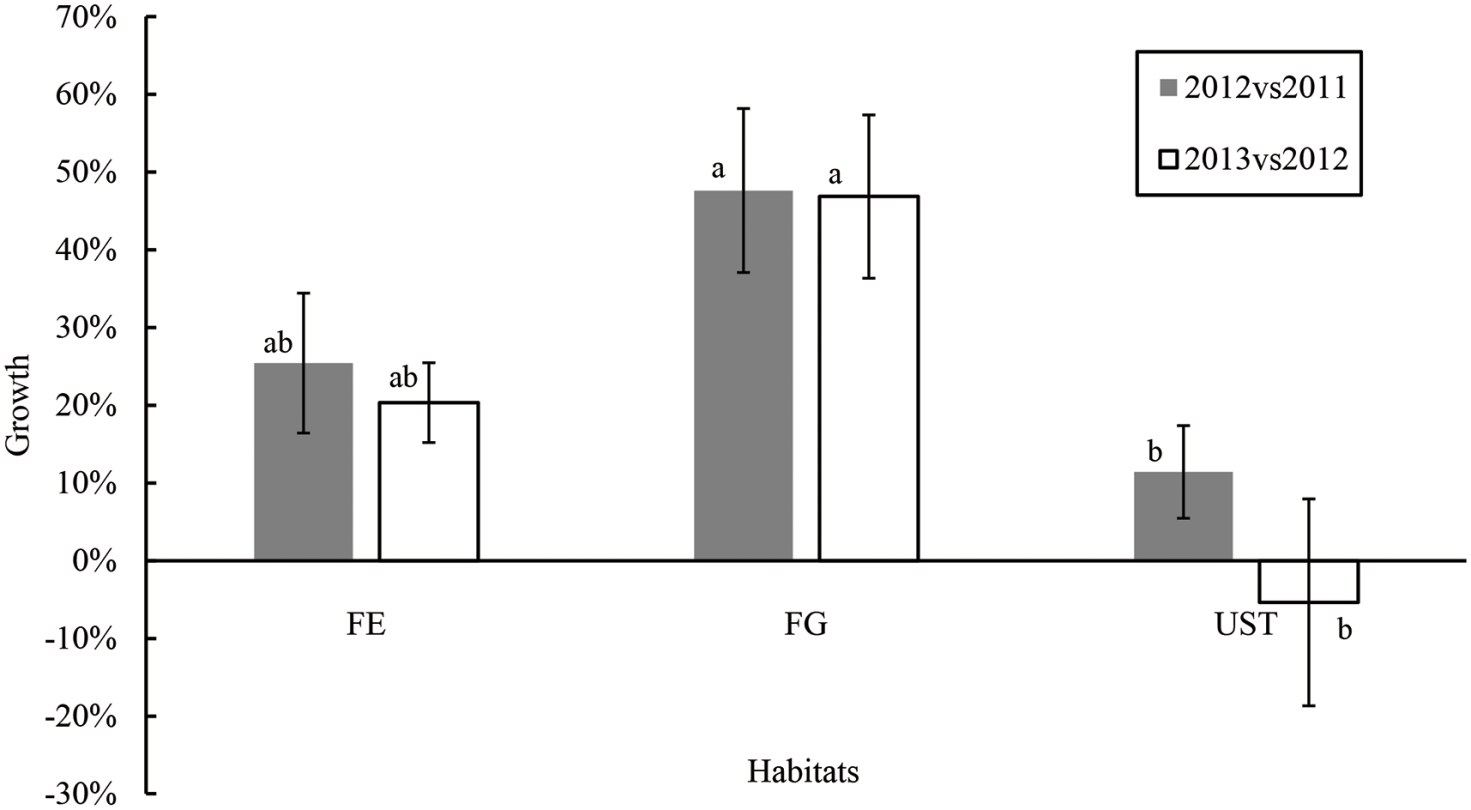
Growth of *C*. *tibeticum* population from 2011 to 2013. Values are the means of three replicates, and the vertical bars represent the SE. Different letters in the same row indicate significant difference (*P* < 0.05).

According to the population surveys from 2011 to 2013, we found that the annual rates of population increase ranged from approximately -5.3% to 47.6% (Fig 9). The general tendency of the increase was highest in FG, median in FE, and lowest in UST. Notably, negative growths occurred in the populations in UST (S3 Table).

## Discussion

### Photosynthesis in the three habitats

The dynamics of *PAR* recorded on several sunny days (Fig 2A and S1 Fig) demonstrated that there were heterogeneous light regimes corresponding to the three habitats. The populations of *C*. *tibeticum* in FE received the highest *PAR* during daytime, followed by those in FG and UST. Since light is one of the most important driving forces of photosynthesis, the diurnal variation in *P*_n_ in these habitats tightly followed the fluctuations in light (Fig 2C). Generally, the orchid in FE had a higher *P*_n_ than those in FG and UST. Notably, *P*_n_ of *C*. *tibeticum* was depressed in FE habitats at approximately 1300 HR. High light and leaf temperature as well as stomatal closure are factors that can induce midday depression of photosynthesis [27]. Our results showed that *g*_s_ (Fig 3C) and *L*_s_ (Fig 3B) in FE had low values at noon, which is when the light intensity and *T*_l_ reached their peak values (approximately 1900 μmol m^-2^ s^-1^, ca. 30°C, respectively; Fig 2A & 2B). A similar phenomenon was also found in *C*. *flavum* under high radiation and leaf temperature [15]. *P*_n_ in FG was low in the early morning, but increased steeply after 1100 HR. This increase was mainly due to direct solar radiation from the high solar altitude and elevated *T*_l_. Under closed canopies, UST plants received a low *PAR* through the day, and this low light environment is the main reason for the lower *P*_n_ observed for UST plants.

Plants of *C*. *tibeticum* in different habitats had distinct photosynthetic capacities. Some of the gas exchange parameters of the populations in the three habitats, including *P*_max_, *CE*, *LSP*, *R*_d_, and *V*_cmax_, decreased progressively from higher light habitats to lower ones (Table 2). Similar results were observed in *C*. *flavum* that naturally occurred in a variety of radiation regimes [8]. The higher photosynthetic capacities of both FE and FG plants were reflected in the higher *LMA* than found in UST. Our results indicated that the *LCP* and *R*_d_ in FG and UST were lower than those in FE. Compared with heliophytes, ombrophytes generally have lower values of *LCP* and *R*_d_ [28], which is a strategy for the latter to improve the carbon balance [15,29,30]. The highest *AQY* of *C*. *tibeticum* in UST indicates that plants in this habitat have the greatest capacity to utilize weak light [31–33].

### Leaf morphological adaptability in different habitats

Heterogeneous habitats with different canopies in the subalpine forest cause varying degrees of light stress in the leaves of *C*. *tibeticum*. The leaf morphology of *C*. *tibeticum* shows considerable phenotypic plasticity in response to different light intensities. A previous study indicated that variations in the leaf traits of orchids enable them to adapt to changing environments [19].

The leaf areas (Table 3) of the populations in FG and UST were higher than those in FE. This result agrees with the theory that plants often adapt to low irradiance by increasing their leaf area [34] to capture more light [35,36]. Conversely, the decrease in leaf area in UST compared with that in FG might be ascribed to the minimal photosynthesis in UST, which limited morphogenesis, compared with that in FG. Moreover, *TtL* and *NTB* in UST are the lowest among these habitats. The decreased *TtL* and *NTB* would increase the use of the diffuse radiation [37,38] and decrease the reflection of direct light, respectively, which are considered to be adaptations to low light environments [39,40].

The growth of *C*. *tibeticum* at FE was depressed in the high light environment, as shown by the lower *PH*, *TtL*, and TM than those in FG. Sun-exposed leaves, compared with shaded leaves, increased the total laminar thickness, which has a role in light capture [41]. By contrast, we speculated that the decrease in *TtL* and *TM* in FE might reduce light interception because of the higher luminousness of the leaves. Generally, a high level of UV-B decreases vascular plant biomass production and other growth parameters [42–46]. The ultraviolet-B (UV-B) radiation dose is high in the subalpine forest [47,48], especially at the forest edge without canopies. The UV-B intensity in FE was extremely high, up to 49.14±3.57 W m^-2^, which was almost approximately 4- and 210- fold greater than those in FE and UST (Table 1 and S2 Table), respectively. Interestingly, compared with other shaded populations, *NTB* (25.09±0.98 cm^-2^) and *NTF* (57.20±1.27 cm^-2^) of the plants in FE were 1.6- and 1.5-fold higher, respectively. High-density hairs on the leaves of plants at a high altitude are UV-acclimation characteristics that can reflect UV-B radiation and retard photo damage [40]. Moreover, the *Fv*/*Fm* of the leave in FE at noon was significantly lower than in the morning and remained low in the afternoon. This might indicate that photosystem II in plants in FE was damaged under full sunlight and high UV-B radiation [49].

### Reproductive variations of populations at different habitats

The flowering- and fruiting- rates of *Cypripedium*’s natural populations varied greatly in different habitats [15]. Light, as an ecological factor, influences the reproductive success of this genus [13,14]. In environments with greater light penetration, *Cypripedium* species will receive higher irradiance and accumulate biomass, which is favorable for growth and reproduction [14]. In this study, the *FR* decreased significantly from the higher to lower light area (Table 3). Our analysis revealed that there were positive correlations, including both *PAR* and *P*_max_ to *FR*, respectively (Fig 7). Higher photosynthetic rates increase the carbon gain and fruit production [15]. However, in the present study, the *FrR* was 11.9% in moderate light habitat (FG), but zero in both FE and UST. The zero-fruit setting in UST might be ascribed to small populations (less than 30 plants generally) with low *FR*s, which resulted from insufficient biomass accumulation (low *LMA*, Table 2). However, a possible explanation of the lack of fruits in FE was the change in the floral function morphology. According to assessments of the floral functional morphology (Table 3 and Fig 6C the *SL* and *AL* of *C*. *tibeticum* in FG and UST were slightly lower than the bumblebee queen’s thorax height (*TH*, 0.71 ± 0.061 cm, mean ± SE) [5], which guarantees that pollinators can easily go through the gallery and pollinate. However, the gallery of flowers in FE was at least 25% lower than the *TH* and too narrow for queens to pass.

The abundance and distribution orchid species are inclined toward FG. According to the three years of observation in the field, plants in FG have the largest population sizes (more than 103 plant per population) and highest growth rate compared to those in FE and UST (Fig 9). Conversely, sexual propagation was only determined in FE colonies, which increases the genetic diversity of the population and reserve the adaptive potential and plasticity[50] to adapt to environmental changes [51,52]. Although the populations in FE had relatively high *FR*, new individuals reproduced by cloning ramets occupies the original colonies and a high population density was found (S2 Fig). Hence, the competition for resources increased, and the scenario was detrimental to development. In UST, the populations had the lowest growth and decreasing trends were also observed (Fig 9), which are not conducive to habitats with low light for the survival of orchids.

Thus, *C*. *tibeticum* can adjust its photosynthetic capacity and leaf traits to maintain a functional balance in response to different environments. The reproduction of this species is profoundly affected by light availability in different habitats of the subalpine forest. Our results showed that neither full- (under FE) nor low- (US) illumination favored the expansion of *C*. *tibeticum*. Our study could serve as a guide for the protection and reintroduction of this species *in situ*, emphasizing the importance of specific light regimes for the expansion of *Cypripedium* sp.

## Supporting information

S1 Fig Diurnal changes of the photosynthetic active radiation (*PAR*, A) during May and September in the different habitats. Each point represents the mean ± SE (n = 3).
(TIF)

S2 Fig Two populations of *C*. *tibeticum* in FE. (A) A population next to *Quercus senescens*; (B) a population surrounded by *Sabina squamata*.
(TIF)

S1 Table Diurnal changes in photosynthetic active radiation (μmol m^-2^ s^-1^) in 2012 and 2014. (XLSX)

S2 Table Records of UV-B radiation (W m^-2^) in the three habitats at noon. (XLSX)

S3 Table Growth of *C*. *tibeticum* populations in each quadrats. (XLSX)
