# Supplementary figures and images for: Photosynthetic, morphological, and reproductive variations in Cypripedium tibeticum in relation to different light regimes in a subalpine forest
Source: PLoS One. 2017 Jul 12;12(7):e0181274. doi: 10.1371/journal.pone.0181274 (PMC5507556; doi:10.1371/journal.pone.0181274)

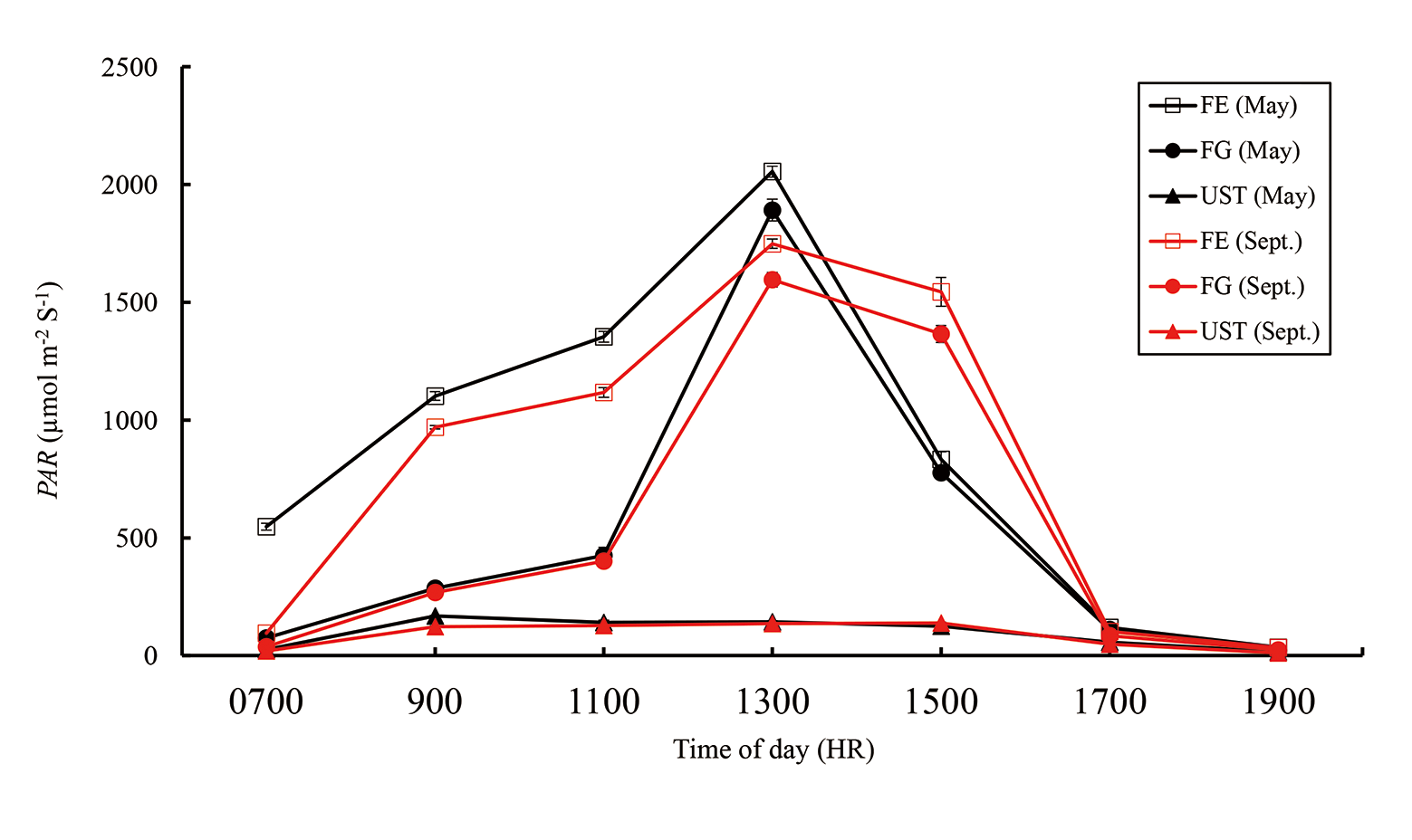

Supplement: S1 Fig — Each point represents the mean ± SE (n = 3). (TIF) [file pone.0181274.s001.tif]

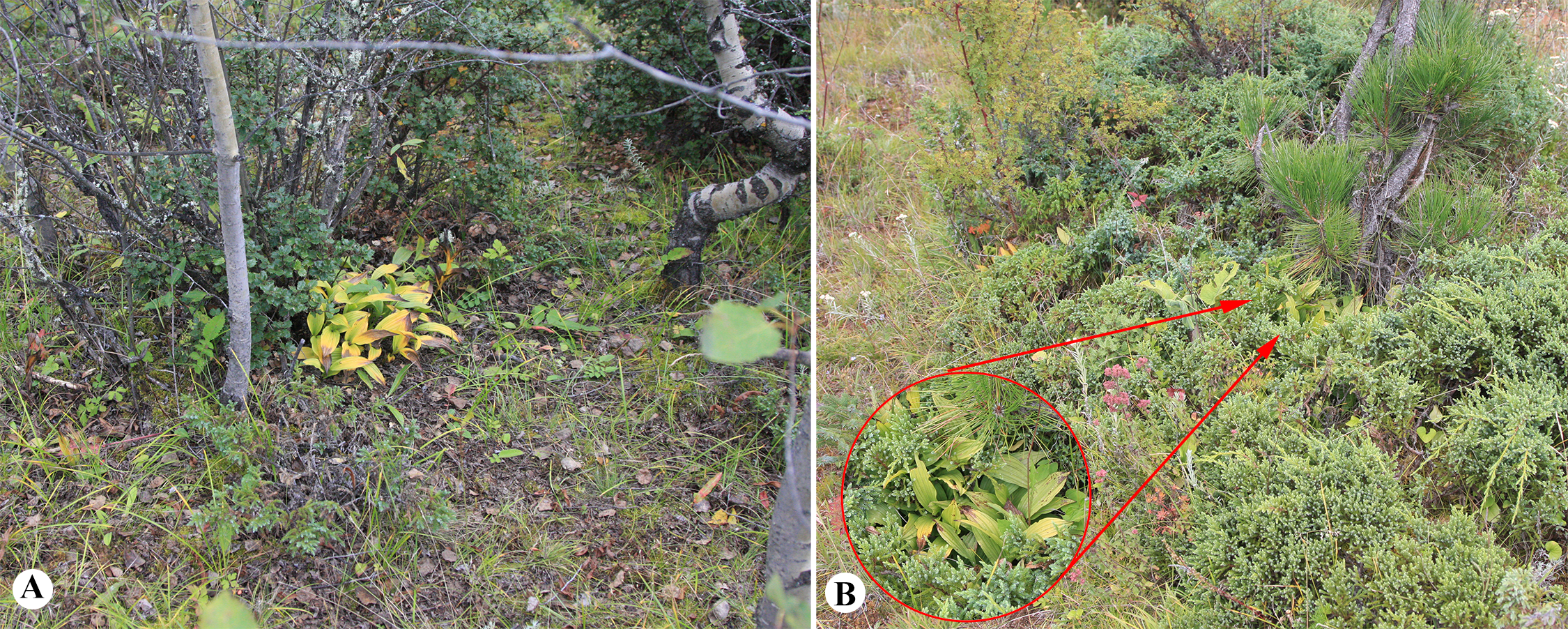

Supplement: S2 Fig — (A) A population next to Quercus senescens; (B) a population surrounded by Sabina squamata. (TIF) [file pone.0181274.s002.tif]
